# Dewetting-Assisted Patterning: A Lithography-Free Route to Synthesize Black and Colored Silicon

**DOI:** 10.1021/acsami.3c08533

**Published:** 2023-09-05

**Authors:** Amin Farhadi, Theresa Bartschmid, Gilles R. Bourret

**Affiliations:** Department of Chemistry and Physics of Materials, University of Salzburg, Jakob Haringerstraße 2a, A-5020 Salzburg, Austria

**Keywords:** metal-assisted chemical etching, silicon nanowires, dewetting, nanostructured silicon, black silicon, colored silicon

## Abstract

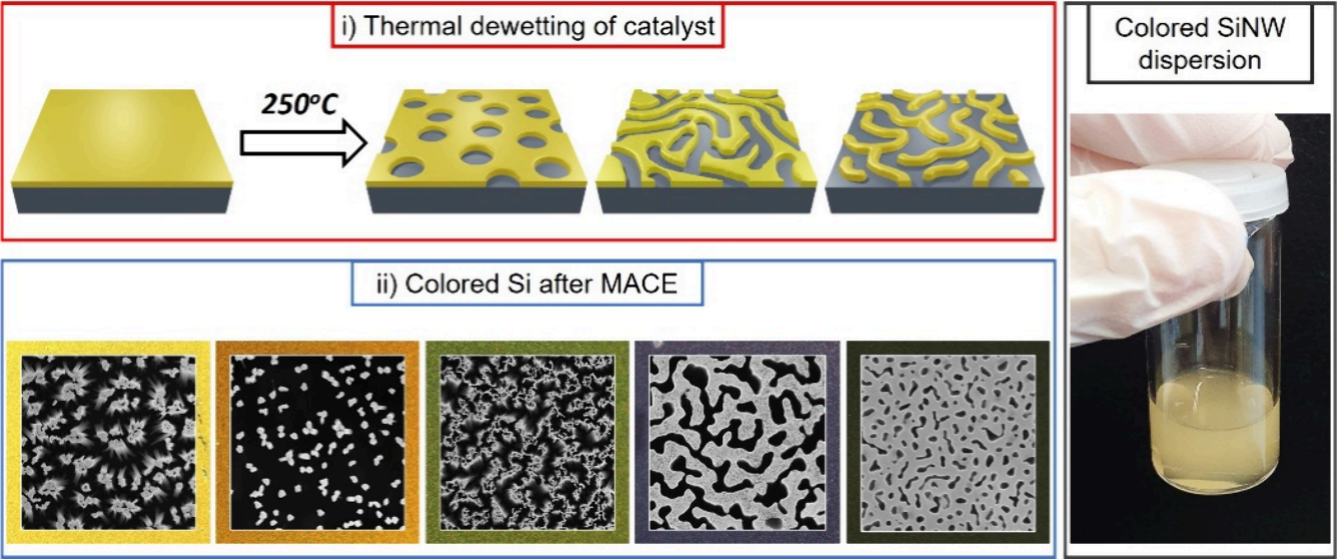

We report the use of thermal dewetting to structure gold-based catalytic etching masks for metal-assisted chemical etching (MACE). The approach involves low-temperature dewetting of metal films to generate metal holey meshes with tunable morphologies. Combined with MACE, dewetting-assisted patterning is a simple, benchtop route to synthesize Si nanotubes, Si nanowalls, and Si nanowires with defined dimensions and optical properties. The approach is compatible with the synthesis of both black and colored nanostructured silicon substrates. In particular, we report the lithography-free fabrication of silicon nanowires with diameters down to 40 nm that support leaky wave-guiding modes, giving rise to vibrant colors. Additionally, micrometer-sized areas with tunable film composition and thickness were patterned via shadow masking. After dewetting and MACE, such patterned metal films produced regions with distinct nanostructured silicon morphologies and colors. To-date, the fabrication of colored silicon has relied on complicated nanoscale patterning processes. Dewetting-assisted patterning provides a simpler alternative that eliminates this requirement. Finally, the simple transfer of resonant SiNWs into ethanolic solutions with well-defined light absorption properties is reported. Such solution-dispersible SiNWs could open new avenues for the fabrication of ultrathin optoelectronic devices with enhanced and tunable light absorption.

## Introduction

Thanks to its high abundance, optimum band gap for solar absorption and tunable electronic properties, silicon has been the material of choice for essential technologies in microelectronics and photovoltaics.^1,2^ Nanostructuring silicon provides unique properties with potential applications in electronics,^3^ photonics,^4−8^ sensing,^9−14^ energy storage^15,16^ and conversion,^5,17−23^ and nanomedicine.^24^ When structured into vertically aligned Si nanowires (VA-SiNW) arrays, Si can sustain a variety of optical resonances, such as Fabry-Pérot and Mie resonances, and leaky waveguide modes.^4−7^ Such VA-SiNW arrays can give rise to functional silicon surfaces with vibrant colors,^4,6^ providing an elegant path toward the fabrication of “colored silicon”, investigated for optical sensing,^13^ and the manufacture of color filters.^6^ Additionally, the high reflectance of bulk silicon surfaces can be suppressed down to a few percent using structured black Si, which is highly relevant for solar conversion systems:^5,17−23^ For example, nonuniform black VA-SiNWs films were used as an antireflection coating with a high angular acceptance to improve the efficiency of state-of-the-art silicon solar cells built with interdigitated back-contacts.^21,22^ Instead, properly nanostructured colored silicon could be used as a wavelength selective absorption layer to produce silicon solar cells with well-defined and vivid colors. Although yet unexplored, such colored silicon could provide another path to engineer the aesthetics of silicon solar cells for colored photovoltaics.^25,26^ Since the optical properties of both black and colored silicon are strongly geometry dependent, finding cost-effective methods to reliably nanostructure silicon is essential.

The fabrication of VA-SiNW arrays can be achieved via bottom-up vapor–liquid solid (VLS) synthesis^27^ and top-down techniques such as reactive-ion etching (RIE)^14,28^ and metal-assisted chemical etching (MACE).^4,29,30^ While VLS can suffer from charge recombination using the conventional gold catalyst,^31^ RIE yields surface damage and requires expensive instrumentation that is not accessible to every research groups.^32^ Instead, MACE has appeared as a versatile, benchtop, solution-phase and cost-effective alternative to structure silicon.^22,23,29,30,33^ MACE uses a nanostructured catalytic metal mask, usually made of gold, to etch through silicon: When immersed into an HF/H_2_O_2_ solution, the gold film catalyzes H_2_O_2_ reduction, injecting holes through the metal/Si Schottky barrier and oxidizing Si that dissolves in presence of HF.^34^ Due to the high reactivity of gold toward the reduction of H_2_O_2_, MACE is highly selective: only the silicon in direct contact with the metal is etched. As such, MACE can be used to prepare high aspect ratio Si nanostructures with a variety of cross-sectional morphologies, set by the metal mask patterns.^30,33,35,36^ Compared with other techniques, MACE offers some flexibility:^5^ For example, it can be combined with KOH etching steps to prepare Si nanowires composed of segments with different diameters.^4,5^ Additionally, the presence of the gold film after MACE can used to perform electrochemical deposition and lithographies to prepare complex hybrid metal–Si structures.^5,9,37,38^ While standard photolithography masks can be prepared to synthesize VA-SiNW arrays with diameters > 400 nm, accessing the smaller dimensions required to prepare colored silicon, which can sustain highly absorbing leaky wave-guided modes, requires more advanced patterning approaches such as electron-beam lithography^6,14^ or colloidal lithography.^29,35,39^ Although metal nanoparticles synthesized on a Si wafer via galvanic exchange can be used to synthesize black silicon composed of ill-defined Si nanostructures,^30^ it cannot be used to prepare isolated VA-SiNW with monodisperse diameters that can support well-defined guided modes.^4,30^

Rather than using complex lithography techniques, we can pattern the metal mask via solid-state dewetting. When prepared via physical vapor deposition, most metal films are metastable and dewet during annealing: Once the atom mobility becomes sufficiently high, atom transport reduces the system surface energy by creating metal islands.^40^ Usually, the dewetting temperature is much lower than the bulk melting temperature,^40^ making dewetting a relatively mild process. Because it involves the solid/solid/gas interface, dewetting requires the presence or the formation of holes and film edges to drive atom transport, and proceeds through different stages: hole formation, hole growth, and film break up.^40^ These solid-state transformations have been used to prepare a variety of metal holey films and metal nanoparticles on flat but also on nanostructured surfaces.^40−42^

To-date, the combination of dewetting-assisted patterning with MACE to synthesize silicon nanowires has only been considered in a multistep process: (i) Ag deposition and film dewetting into nanoparticles, (ii) gold film deposition, and (iii) lift-off resulting in a holey gold film that can be used for MACE.^43^ This multistep approach is limited by the difficulty in efficiently removing the Ag nanoparticle mask during lift-off due to their small sizes. This issue can be alleviated by preparing the particle mask using Ni instead of Ag.^44,45^ However, much higher temperatures, i.e., >850 °C, are required for dewetting nickel, which requires the formation of a well-defined SiO_2_ barrier layer to protect the silicon during annealing. This complicates the approach, and is not necessarily compatible with the use of standard silicon wafers.^44,45^ Furthermore, the synthesis of well-defined SiNW arrays that can sustain leaky guided modes with tunable light absorption has not yet been demonstrated using nonlithographic methods, which includes the combined thermal dewetting/MACE approach.

**Scheme 1:**
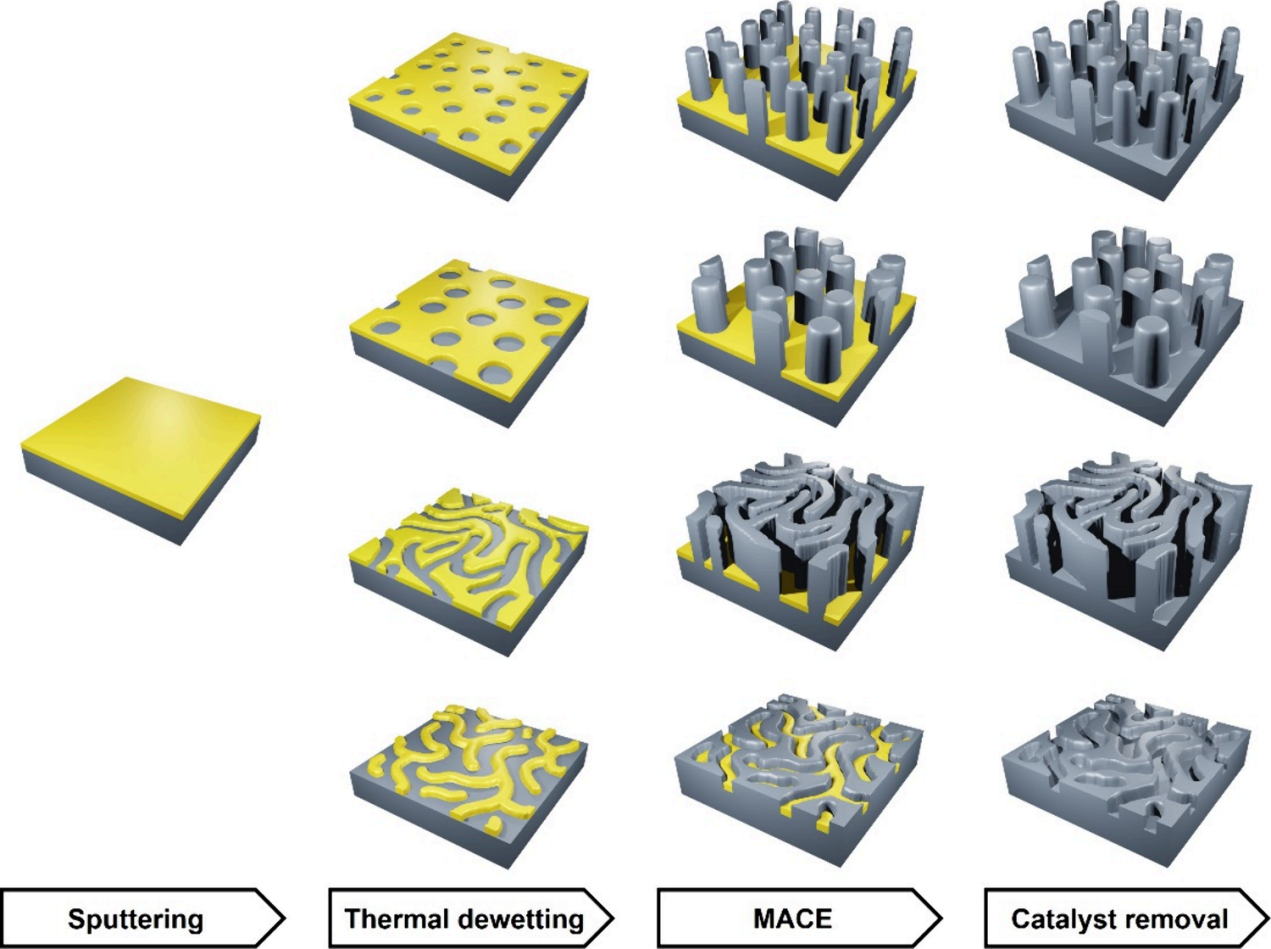
Schematic Illustration of Combined Dewetting-Assisted Patterning and MACE The process starts with sputtering of a catalyst film (yellow) onto the silicon wafer (gray). Annealing at 250 °C leads to dewetting-assisted patterning of the metal catalysts. The catalyst morphologies and dimensions can be tuned by adjusting catalyst thickness, composition and annealing duration. After MACE, the metal catalyst pattern is transferred into silicon, yielding nanostructured silicon with tunable dimension and morphology after the catalyst removal.

Herein, we report the dewetting-assisted patterning of metal films for nanostructuring silicon via MACE (Scheme 1). Au and AuAg films were dewetted on p-type silicon surfaces at 250 °C for various durations, yielding nanostructured metal holey masks with a metal coverage tunable from 98% to 26%, a hole density tunable from 4/μm^2^ to 197/μm^2^, and either circular or serpentine-like hole morphologies. The metal pattern can be transferred into the silicon substrate via MACE to synthesize a variety of nanostructured silicon substrates such as Si nanotubes and nanowalls with thickness tunable between ca. 8 and ca. 140 nm, and Si nanowires that strongly interact with light, with diameters tunable between 40 and 200 nm. The relative size distribution of the nanostructured silicon lateral dimensions is typically below 30%, which demonstrates the uniformity of the structures produced in this work. This is further confirmed by the defined optical resonances and colors observed on some of the samples synthesized here. Additionally, the successful transfer of resonant SiNWs with diameters down to 40 nm into ethanolic solutions is demonstrated. This is, to our knowledge, the first report of SiNW solutions with defined and tunable light absorption in the visible range that are not attributed to quantum sized-effects.

## Experimental Methods

### Materials

All chemicals and solutions listed here were used without further processing, unless noted otherwise. Iodine (99.8%) and potassium iodide (99.99%) were purchased from Sigma-Aldrich. Acetone (99%), ethanol (96%), isopropanol (IPA) (≥98%) and Hydrofluoric acid (40%), hydrogen peroxide (30%) and nitric acid (65%) were acquired from VWR. The water used was double deionized using a MilliQ system with a resistivity of 18 MΩ. P-doped Silicon wafer (50.8 mm, ⟨100⟩, resistivity 1–30 Ω cm, thickness: 275 ± 25 μm) were purchased from Si-Mat, Germany. The TEM grid used for shadow-masking was a 400 mesh Au lacey carbon grid.

### Dewetting-Assisted Patterning of the Catalytic Mask

p-Type Silicon wafers were cut into 1.5 × 1.5 cm^2^ pieces. Similarly to our previous work,^29^ the samples were cleaned via sonication in acetone for 5 min and then treated by oxygen plasma for 5 min (Quorum Emitech K1050X, 50 W, oxygen flow 30 mL min^–1^). Afterward, the Au and Au/Ag films were sputtered onto the cleaned Si substrates using a Cressington Sputter Coater 108 auto at 40 mA for different durations. We observed some differences in the deposition rate depending on the sample location in the Cressington sputtering chamber. To obtain more reproducible Au film morphologies/thicknesses, we advise one to only sputter one sample at a time, located in the center of the deposition chamber. The metal film thicknesses *d*_*Au,Ag*_ were estimated by performing SEM analysis of the metal/Si cross-sections, prepared by cleaving the metal/Si substrates. Because some of the films were porous, the effective metal film thickness *h*_*Au,Ag*_ was used to characterize all films prepared, calculated according to: *h*_*Au,Ag*_ = *d*_*Au,Ag*_ × *X*_*Au,Ag*_ where *X*_*Au,Ag*_ is the corresponding metal film coverage, calculated using top-view SEM images and ImageJ. The standard deviation of the thickness measurement is provided for each sample and is typically 1–2 nm since most films are flat. In order to pattern the catalyst mask, the samples were loaded into a hot Nabertherm ashing furnace (model L 15/11) that was preheated to 250 °C and annealed at 250 °C for a duration t_A_ ranging from 30 s to 30 min. The samples were then removed from the furnace and left to cool down to room temperature.

To prepare samples with Au film on AZO/Si, a thin adhesion layer of Al-doped ZnO was sputtered directly onto the Si substrates before the Au deposition, using a Clustex 100 M sputtering system from Leybold Optics, as previously described.^29^ In short, sputtering was performed for 1 s using Argon gas at a pressure at 3 × 10^–3^ mbar at 75 W.

### Fabrication of the Si Nanostructures via Metal-Assisted Chemical Etching (MACE)

MACE was performed similarly to our previous works.^29,37^ In short, metal coated-Si substrates were immersed in a MACE solution containing 10 mL of H_2_O, 10 mL of HF, and 1 mL of H_2_O_2_ for various durations, and rinsed three times in MilliQ water. The samples were then cleaned in a 20 mL of H_2_O and 4 mL of HF mixture for 5 min to remove any residual porous Si/SiO_2_ that can be present at the Si nanostructure surface after MACE. After rinsing the samples three times in Milli-Q water and once in ethanol, the samples were dried in air.

SiNW ethanolic dispersions were obtained by scratching the Si substrates with a tweezer, followed by 30 min sonication in ethanol. Visual inspection of the samples after this treatment suggests very efficient transfer of the SiNWs into solution.

### Patterning via Shadow-Masking

Colored Si patterns were prepared via shadow masking using a TEM grid (lacey carbon film on Au carrier mesh, mesh 400) as a mask. First, the p-type Si slide was cleaned by sonication for 5 min in acetone, followed by 5 min of OPE at 50 W, before Au was sputtered. The carbon film on top of the Au TEM grid was etched for 5 min in He plasma at 100 W (starting pressure 0.06 mbar), followed by a 10 min OPE treatment at 50 W to ensure full combustion of the carbon layer. The location of the cleaned Au TEM grid was fixed on top of the Si during the second sputtering step: The grid was sandwiched between the Si slide and a thick stainless steel plate perforated with a 2.5 mm circular hole in its center, where the TEM grid was pressed. Ag was then sputtered. This shadow masking step can be used to pattern the Au-coated Si slides with Ag within micrometer sized square regions. The patterned metal films were then annealed for 5 min at 250 °C. MACE was performed for 2 min in an aqueous solution of HF and H_2_O_2_ (H_2_O:HF:H_2_O_2_ = 10 mL:10 mL:1 mL) followed by a HF after-treatment for 5 min (HF:H_2_O = 4 mL:20 mL). Au was dissolved for 2 h in an aqueous KI/I_2_ solution (10 wt % KI, 5 wt % I_2_) and Ag in a subsequent etching in a 1:1 solution of H_2_O:HNO_3_ for 2 h.

### Microscopy

Scanning electron microscopy (SEM) images were acquired using a Zeiss Ultra Plus 55. Imaging was performed at an accelerating voltage of 5 kV, with an InLens secondary electron (SE) detector and a working distance of 4 mm. Energy dispersive X-ray spectroscopy (EDS) elemental maps were acquired using a 50 mm^2^ silicon drift EDS detector from Oxford instruments. The freely available software ImageJ was used for size analysis and quantification of the metal catalyst coverage. A Leica S8 APO stereo microscope was used at a magnification of 80× for obtaining the Si substrate light microscopy photographs. Zeiss Axio Imager M2m using a 100× objective was used for imaging the patterned silicon samples.

### Total Reflectance UV–vis Spectra

Total reflectance spectroscopy was carried out using a PerkinElmer Lambda 1050 equipped with a 150 mm integrating sphere and PbS and InGaAs detectors, collecting both the diffuse and the specular reflectance. The catalyst film present on the Si surface after MACE was removed before the reflectance measurements: Pure Au films were etched in a KI/I_2_ solution (10 wt. % KI, 5 wt. % I_2_) for 2 h, and Au/Ag films were etched first in the same KI/I_2_ solution for 2 h and then in a 1:1 H_2_O:HNO_3_ solution for 2 h. A 5 mm circular pinhole was used to select the area of interest. The baseline correction was performed after aligning the light beam on the pinhole by using a white Spectralon reference. The reflectance spectra were recorded between 200 and 1200 nm with a 2 nm step.

### Electromagnetic Simulations with the Finite-Difference Time-Domain (FDTD) Method

The experimentally obtained results were supported by simulating reflectance spectra of the respective samples by using the FDTD Solutions package from Lumerical (Ansys, Inc.). MACE usually leads to a slight tapering of the SiNW, where the top is slightly narrower than the bottom of the wire due to the slow dissolution of bare Si in the MACE solution.^4^ Similarly to our previous work on VA-SiNW arrays prepared via MACE, this was taken into account by simulating slightly tapered nanowires arranged in a hexagonal array (pitch: 480 nm, and NW length: 1.5 μm). Such tapering reduces the contribution of the Fabry-Pérot resonances to the simulated reflectance spectra. The bottom nanowire diameter was 10 nm larger than the wire diameter at the top, set to the nominal diameter of 40, 68, and 129 nm, respectively. A 3 nm thin shell of SiO_2_ was added at the surface of the SiNWs in order to account for the native oxide layer forming on Si when exposed to air. All simulated SiNW structures were simulated in air (a surrounding medium with a dielectric constant of 1). The Si and SiO_2_ dielectric functions were used directly from the Lumerical materials library which is part of the software package (data from Palik).^46^ A mesh size of 4 nm was used around the SiNWs. Light was injected along the *z*-axis using a plane wave source in the wavelength range between 300 and 1000 nm. The hexagonal geometry allowed the use of symmetry boundary conditions that were set to antisymmetric along the *x*-axis (polarization direction of the electric field component) and symmetric along the *y*-axis. A reflectance monitor was placed behind the light source in order to collect the light reflected from the arrays.

## Results and Discussion

Prior to thermal dewetting and MACE, metal films were sputtered on clean *p*-type Si (100) substrates. Different films were studied: Au on Al-doped zinc oxide (AZO), pure Au, and bilayer Au/Ag films.

### Au/AZO Films

Our standard metal film preparation involves the sputtering of a thin AZO adhesion layer to improve the stability of the sputtered gold film during MACE,^29^ which is patterned via colloidal lithography.^29^ The possibility of controlling the Au/AZO film nanoscale morphology without a patterning step was investigated by sputtering Au on top of AZO on the flat Si substrate for different times (Figure S1). At low film thickness, i.e., low gold sputtering time *t*_S_ = 10s, isolated and closely spaced small Au nanoparticles form (diameter around 8 ± 3 nm). These small nanoparticles are unstable and do not etch silicon in an anisotropic manner under our standard MACE conditions, yielding mesoporous silicon after MACE with 12 ± 6 nm pore sizes. Slightly longer sputtering (*t*_S_ = 20 s) results in the formation of thin serpentine-like and interconnected metal lines. After MACE, such metal films yield Si tubular structures with a wall thickness of ca. 7 ± 1 nm. Longer sputtering durations yields nonporous continuous metal films that cannot be used for MACE: Such films are not able to provide appropriate mass-transport, which is crucial for MACE.^29^

### Au Film Dewetting on Si

Dewetting-assisted patterning was investigated on gold films sputtered directly on silicon (i.e., without an AZO adhesion layer), and annealed at 250 °C. At low film effective thickness *h*_Au_, e.g., *h*_Au_ < 10 ± 1 nm, typically obtained at *t*_S_ < 60s, a porous serpentine-like Au film results (Figure S2a). At intermediate thickness, e.g., 11 ± 1 nm ≥ *h*_Au_ ≥ 10 ± 1 nm, typically obtained for 70 s ≥ *t*_S_ ≥ 60 s, holey Au films result, with a hole density tunable from ca. 14/μm^2^ to ca. 340/μm^2^ (Figure S2b-d). At *h*_Au_ ≥ 12 ± 1 nm, obtained at *t*_S_ ≥ 80s, continuous Au films are obtained (Figure S2e). Thinner films, e.g., *h*_Au_ < 8 ± 1 nm, dewetted within seconds to produce isolated Au NPs, which are incompatible with the synthesis of anisotropic structures via MACE. Dewetting continuous Au films prepared with *t*_S_ ≥ 80s was not possible under our experimental conditions, even after longer annealing times. Such films were thus not investigated for MACE. Additionally, without an AZO adhesion layer and without annealing, the as-sputtered gold films could not be used for MACE: Extensive and frequent delamination occurred under our MACE experimental conditions.^29^ However, short thermal annealing/dewetting greatly improved the Au film adhesion, which could then be used for performing MACE appropriately. In general, longer annealing times were the most efficient at preventing metal film delamination during MACE.

**Figure 1:**
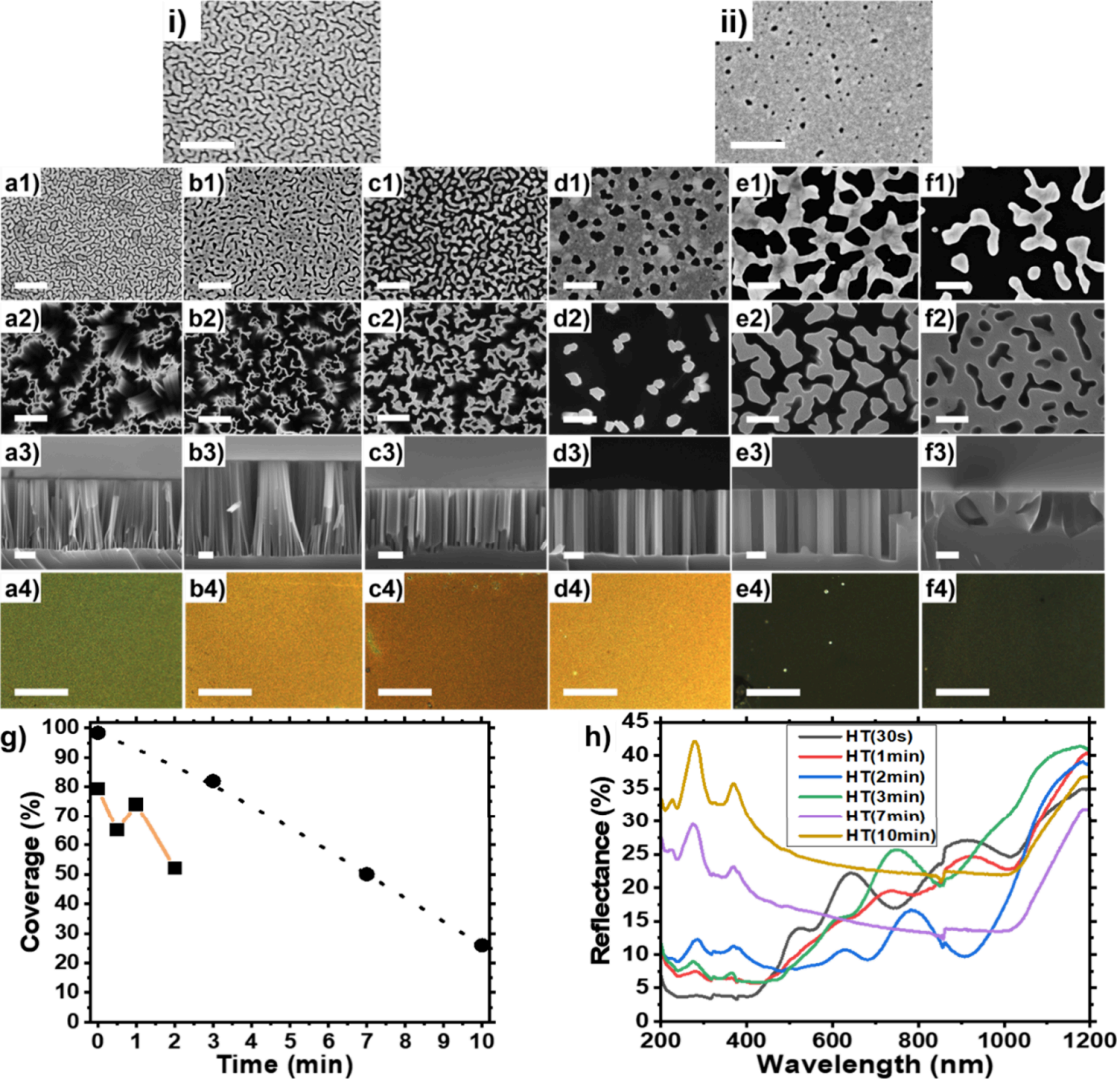
Top-view SEM images of the as-sputtered Si substrates with effective gold film thickness of (i) *h*_Au_ ∼ 9 ± 1 nm (serpentine-like morphology) and (ii) *h*_Au_ ∼ 11 ± 1 nm (holey film), used for thermal-dewetting during different annealing times *t*_A_ at 250 °C and MACE. Effective gold film thickness used to prepare samples shown in (a–c): *h*_Au_ ∼ 9 ± 1 nm and samples shown in (d–f): *h*_Au_ ∼ 11 ± 1 nm. (a1–a4) *t*_A_ = 30 s, (b1–b4) *t*_A_ = 1 min, (c1–c4) *t*_A_ = 2 min, (d1–d4) *t*_A_ = 3 min, (e1–e4) *t*_A_ = 7 min, and (f1–f4) *t*_A_ = 10 min. (a1–f1): Top-view SEM images of the Au film after annealing; scale bars: 200 nm. Top-view (a2–f2) and cross-sectional (a3–f3) SEM images of the resulting silicon nanostructures after MACE, scale bars: 200 nm. (a4–f4) Optical microscopy images the silicon nanostructures synthesized via MACE, scale bars: 100 μm. (g) Au film coverage as a function of annealing time for both types of films: black squares and orange line: *h*_Au_ ∼ 9 ± 1 nm, and black circles and dotted black line: *h*_Au_ ∼ 11 ± 1 nm. (h) Reflectance spectra of the respective nanostructured silicon substrates prepared after annealing Au films with *h*_Au_ ∼ 9 ± 1 nm for 30s (black), 1 min (red), 2 min (blue), and Au films with *h*_Au_ ∼ 11 ± 1 nm for 3 min (green), 7 min (purple), and 10 min (yellow).

Two Au film thicknesses were investigated for nanostructuring silicon via MACE **(**Figure 1): serpentine-like porous Au films with *h*_Au_ ∼ 9 ± 1 nm (Figure 1i, a–c), and slightly thicker holey Au films (*h*_Au_ ∼ 11 ± 1 nm) with a hole density of ca. 240/μm^2^ (Figure 1ii, d–f). Figure 1 shows the influence of thermal annealing duration at 250 °C on the two types of gold films investigated and the resulting silicon morphologies produced after MACE. At *h*_Au_ ∼ 9 ± 1 nm, dewetting occurs within seconds: The voids present in the percolated serpentine-like gold film morphology increase in size for 1 min ≥ *t*_A_ ≥ 30s (Figure 1a1, b1). After two min of annealing, a discontinuous porous gold film is obtained (Figure 1c1).

The gold holey mesh (*h*_Au_ ∼ 11 ± 1 nm), on the other hand, is more stable and barely changes within that time frame (results not shown). At *t*_A_ = 3 min, larger circular holes result from dewetting (Figure 1d1), which increase in size with longer annealing (*t*_A_ = 7 min; Figure 1e1). At *t*_A_ ≥ 10 min, the Au film finally dewets into isolated large Au islands (Figure 1f1). Table 1 summarizes the morphology and dimension of the various nanostructured silicon samples prepared in this work after MACE: tubular Si structures with a wall thickness tunable from 8 ± 2 nm, to 10 ± 2 nm and 17 ± 4 nm can be synthesized by annealing the serpentine-like Au film (*h*_Au_ ∼ 9 ± 1 nm) for *t*_A_ = 30s, *t*_A_ = 1 min, and *t*_A_ = 2 min, respectively (Figure 1a-c). After *t*_A_ = 3 min and MACE, the thicker holey Au film (*h*_Au_ ∼ 11 ± 1 nm) yields monodisperse SiNWs with a diameter of 68 ± 18 nm (Figure 1d). Further annealing for 7 min yields a gold mesh perforated with irregularly shaped holes, which after MACE produces isolated wall-like structures with undefined shapes and a thickness of 74 ± 20 nm (Figure 1e). The isolated gold islands that result after *t*_A_ = 10 min etch silicon in a nonanisotropic fashion (Figure 1f). Overall, the nanostructured silicon coverage can be adjusted from 18% to 74% by adjusting the Au film thickness and annealing time. Vivid colors were observed for *t*_A_ ≤ 3 min (Figure 1a4–d4), which demonstrates the viability of the approach to synthesize colored nanostructured silicon, e.g., green, orange, brown, or black.^4^ The oscillations seen in the UV–vis are likely to be due to Fabry-Pérot resonances.^29,47^ Longer annealing of the 11 ± 1 nm thick Au film (*t*_A_ ≥ 7 min) produced irregularly structured silicon, yielding black samples with reduced reflectance in the 200–1200 nm range. Because of the short annealing times used to prepare the tubular silicons (*t*_A_ ≤ 2 min, Figure 1a–c), a rapid thermal annealing (RTA) setup might be better suited than the standard ceramic furnace used in this work to prepare these samples. Alternatively, a lower annealing temperature could be used to obtain a more controlled dewetting^40,48^ with longer annealing times that are more compatible with the use of a standard furnace.

**Table 1:** Morphology, Dimension, Si Nanostructure Coverage, and Color of the Different Si Substrates Prepared via Dewetting-Assisted Patterning and MACE.

Film thickness and composition^a^	Morphology	Catalyst annealing time *t*_A_ [min]	Si nanostructure dimension^b^ [nm]	Si nanostructure coverage [%]^c^	Color
Au: *h*_Au_ ∼ 9 ± 1 nm	tubular	0.5	*w* = 8 ± 2	35.2	Green
		1	*w* = 10 ± 2	25.4	Orange
		2	*w* = 17 ± 4	48.1	Orange
Au: *h*_Au_ ∼ 11 ± 1 nm	wire	3	*d* = 68 ± 18	18	Orange
	wall	7	*w* = 74 ± 20	49.3	Black
	wall	10	*w* = 69 ± 17	74	Black
AuAg: *h*_Au,Ag_ ∼ 19 ± 2 nm	wire	5	*d* = 40 ± 12	14.1	Yellow
	wall	7	*w* = 44 ± 11	35	Black
		10	*w* = 56 ± 14	54.8	Black
		15	*w* = 64 ± 17	52.6	Black
AuAg: *h*_Au,Ag_ ∼ 44 ± 5 nm	wire	10	*d = 78* ± 23	2	Green
		15	*d*_*a*_ = 129 ± 28	5	Green
		20	*d*_*a*_ = 145 ± 45	11.8	Brown
	wall	30	*w* = 137 ± 32	55.6	Purple

a *h*_Au,Ag_*:* Effective total metal film thickness.

b *w*: wall thickness, *d*: wire diameter, *d*_*a*_: equivalent diameter extracted from the wire cross-sectional area to account for the noncylindrical morphology of these wires.

c Nanostructured silicon coverage estimated as 100% – Au coverage (in %).

### Au/Ag Film Dewetting on Si

**Figure 2:**
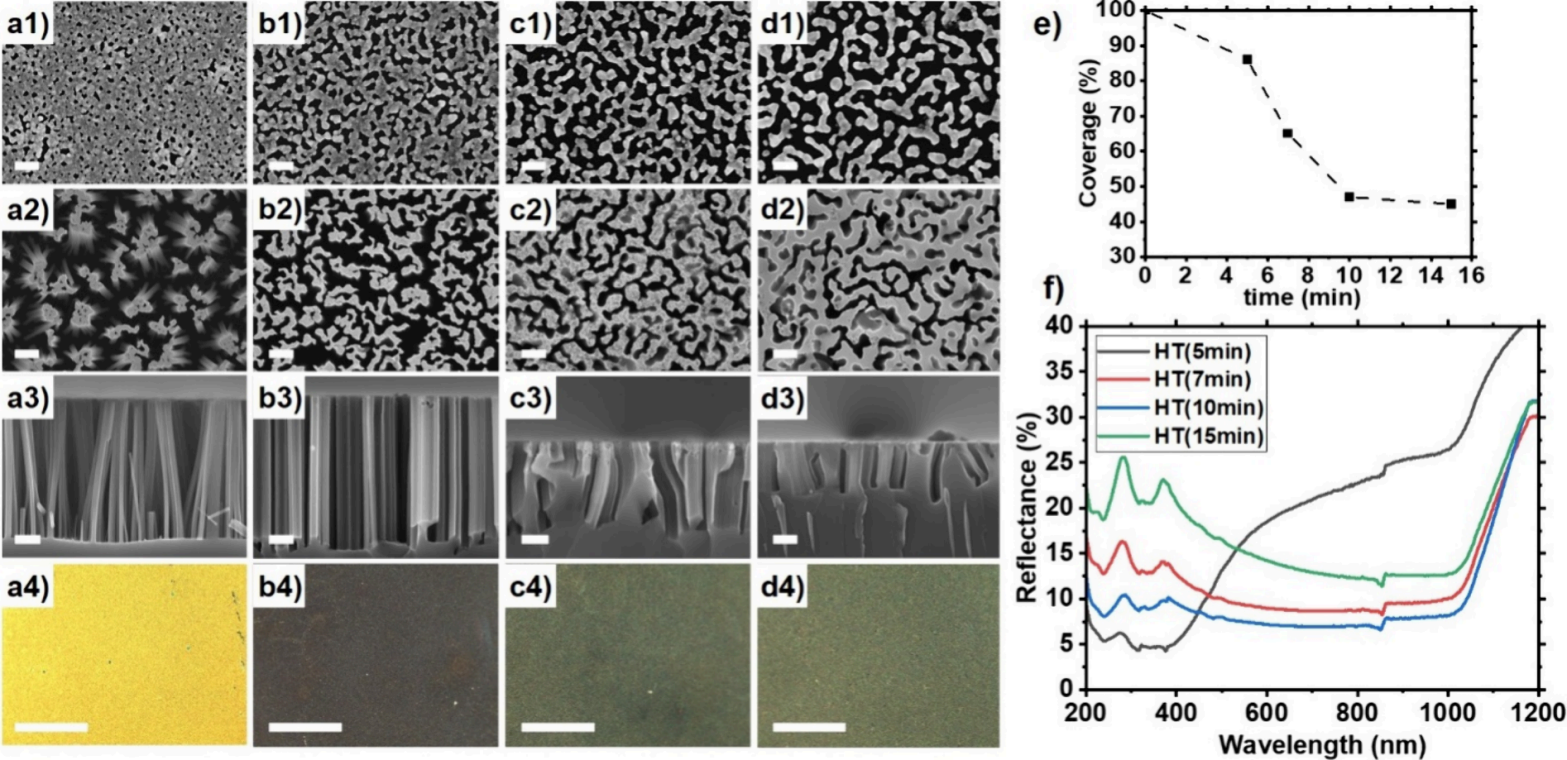
Nanostructured silicon produced after thermal-dewetting of Au–Ag bilayer films with total film thickness *h*_Au,Ag_ ∼ 19 ± 2 nm, prepared with *t*_S_(Au) = *t*_S_(Ag) = 50 s, and two min of MACE: (a1–a4) 5 min, (b1–b4) 7 min, (c1–c4) 10 min, and (d1–d4) 15 min of annealing at 250 °C. (a1–d1): Top-view SEM images of the Au-Ag film after annealing, scale bars: 200 nm. Top-view (a2-d2) and cross-sectional (a3–d3) SEM images of the resulting silicon nanostructures after MACE, scale bars: 200 nm. (a4–d4) Optical microscope images capturing the realistic color of the fabricated silicon nanostructures via MACE, scale bars: 100 μm. (e) AuAg film coverage as a function of annealing time. (f) Reflectance spectra of the nanostructured silicon substrates prepared using AuAg films annealed for 5 min (black), 7 min (red), 10 min (blue), 15 min (green).

The influence of an additional Ag layer was investigated to tune the film morphology further (Figure 2). Aluminum can considerably stabilize Au films during thermal dewetting,^49^ and a similar effect was observed by sputtering a Ag film (*t*_S_ = 50s) on top of a Au film (*t*_S_ = 50s), which delays dewetting. Without annealing, porous AuAg films form (Figure S3), with a total approximate effective film thickness of *h*_Au,Ag_ ∼ 19 ± 2 nm. After five min of annealing, the AuAg film dewets, yielding a highly porous holey mesh (hole density of 197/μm^2^). After MACE, a dense array of vertically aligned thin SiNWs with a diameter of ca. 40 ± 12 nm (Figure 2a) is obtained, which selectively absorb light around ca. 380 nm and have a characteristic bright yellow color. EDS mapping suggests that mixing between the Au and Ag films quickly occurs during thermal annealing, which thus dewets into a AuAg alloy (Figure S4-S5). Further annealing (*t*_A_ ≥ 7 min), leads to a serpentine-like metal catalyst film, which after MACE produces irregular wall-like Si nanostructures with a thickness adjustable in the 44–64 nm range (Figure 2b–d). Due to the large dimensions and irregularity of the silicon nanostructures produced with *t*_A_ ≥ 7 min, these samples are black and dark green. Average reflectance values down to 7.5–10% were measured at *t*_A_ = 10 min. Overall, the annealing duration *t*_A_ can be used to adjust the nanostructured silicon coverage obtained after MACE between ca. 14% and 55%.

**Figure 3:**
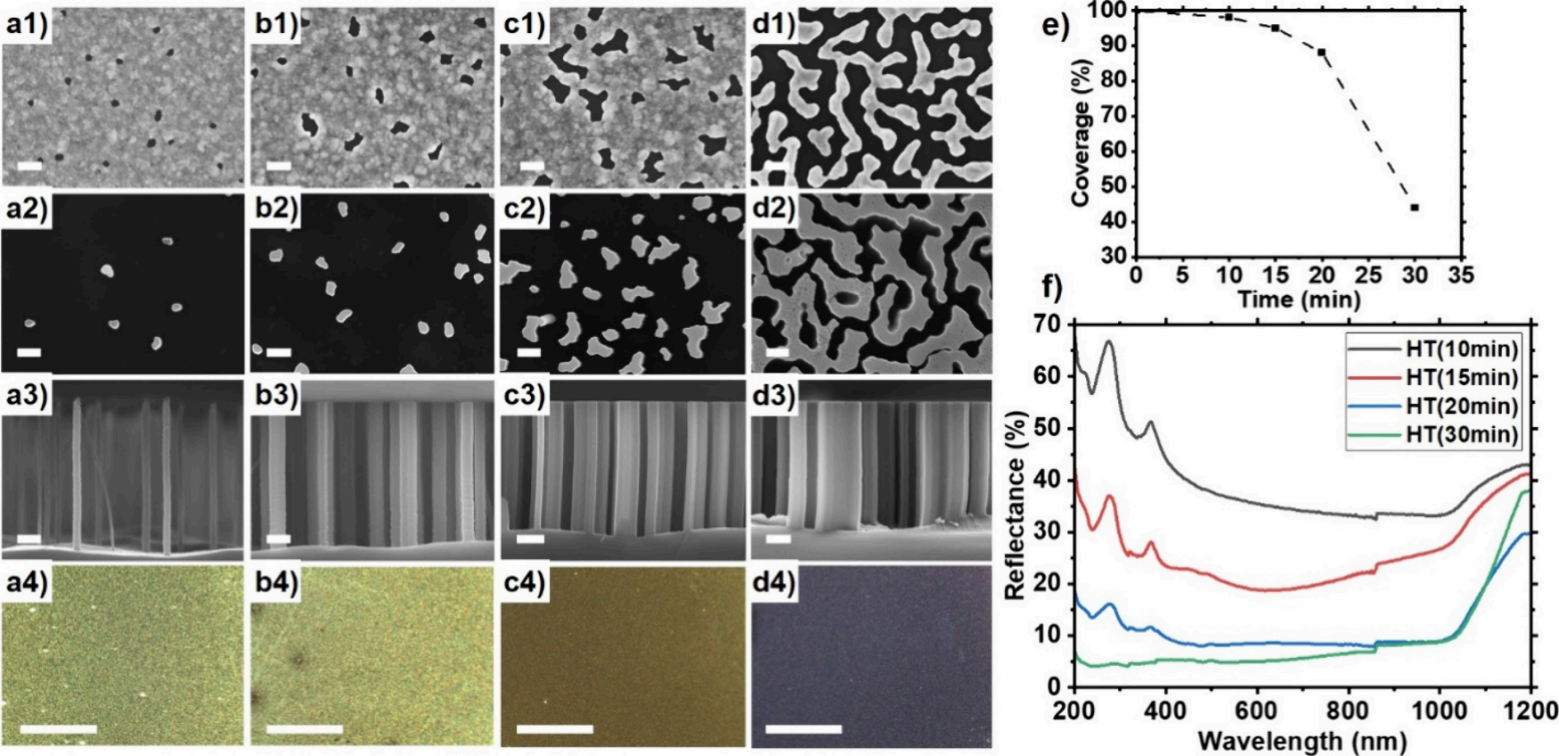
Nanostructured silicon produced after thermal-dewetting of Au-Ag bilayer films with total film thicknesses of *h*_Au,Ag_ ∼ 44 ± 5 nm, prepared after sputtering Au and Ag for 70 s (i.e., *t*_S_(Au) = *t*_S_(Ag) = 70 s) and six min of MACE. (a1–a4) 10 min, (b1–b4) 15 min, (c1–c4) 20 min, (d1–d4) 30 min of annealing at 250 °C. (a1–d1): Top-view SEM images of the Au-Ag film after annealing, scale bars: 200 nm. Top-view (a2–d2) and cross-sectional (a3–d3) SEM images of the resulting silicon nanostructures after MACE, scale bars: 200 nm. (a4–d4) Optical microscope images capturing the realistic color of the fabricated silicon nanostructures via MACE, scale bars: 100 μm. (e) AuAg film coverage as a function of annealing time. (f) Reflectance spectra of the nanostructured silicon substrates prepared using AuAg film annealed for 10 min (black), 15 min (red), 20 min (blue), 30 min (green).

The influence of bimetallic film thickness on dewetting was investigated further by sputtering Ag and Au for longer times (*t*_S_ = 70s for both metals; Figure 3). In this case, nonporous AuAg films are obtained, composed of randomly distributed protrusions (ca. 18 ± 5 nm thick) on a ca. 26 ± 2 nm thick continuous film, with a total film thickness *h*_Au,Ag_ ∼ 44 ± 5 nm. As expected, an increased metal film thickness improves film stability: Thermal dewetting is observed only after *t*_A_ ≥ 10 min, which, after MACE, leads to nanostructured silicon with coverages tunable from ca. 5% to ca. 56% depending on the annealing time. At *t*_A_ = 10 min, a low density of holes forms in the catalyst film, which, after MACE, yields a sparsely dense array of VA-SiNWs with a diameter of ca. 78 ± 23 nm (Figure 3a). The hole size and density increase with longer annealing times (i.e., 20 min ≥ *t*_A_ ≥ 10 min), leading to denser VA-SiNW arrays with large diameters of *d* = 129 nm (*t*_A_ = 15 min, Figure 3b) and *d* = 145 nm (*t*_A_ = 20 min, Figure 3c). At *t*_A_ = 30 min, the catalyst film develops a characteristic serpentine-like morphology (Figure 3d). After MACE, Si nanowalls with thickness of ca. 137 ± 32 nm result, which produce a deep purple color that significantly suppresses reflection: This substrate provides the lowest reflectance reported in this work, with an average total reflectance (i.e., specular + diffuse reflectance) of ca. 5% in the 200–1000 nm range.

### Colored SiNW Substrates

**Figure 4:**
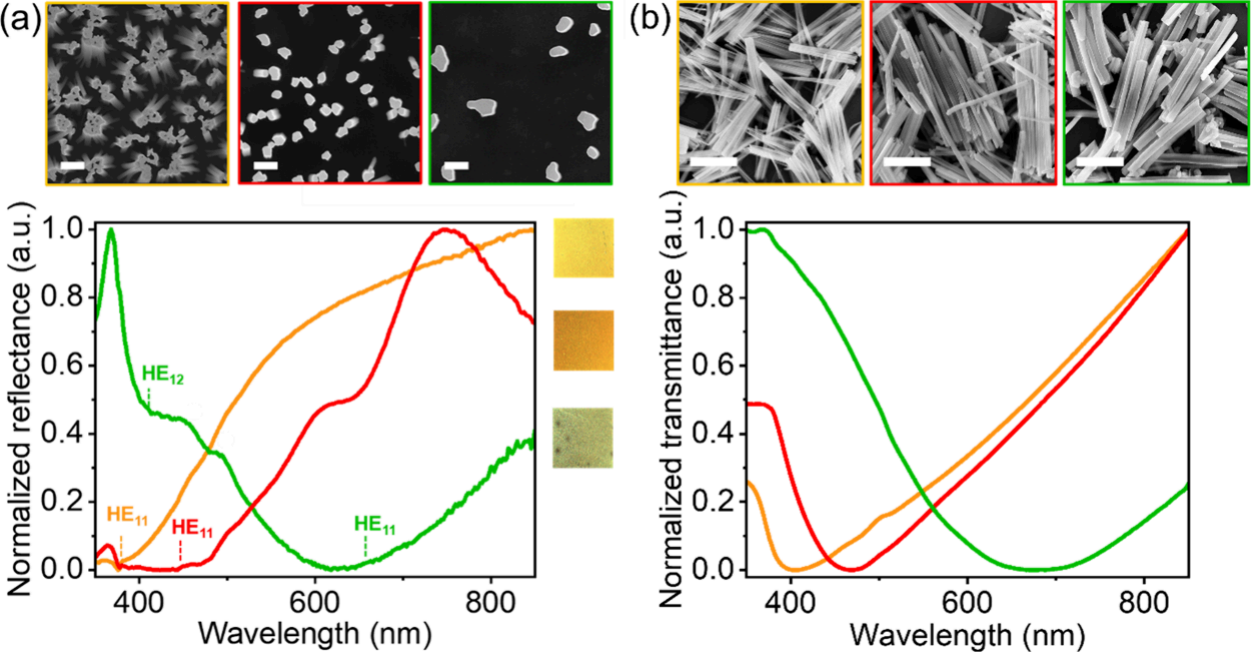
Colored SiNWs prepared via dewetting-assisted patterning and MACE. (a) VA-SiNW arrays. Top, SEM images (scale bars: 200 nm). Bottom, normalized reflectance spectra of various SiNWs: Orange outline and orange curve, SiNWs with a diameter *d* = 40 ± 12 nm, synthesized using a AuAg film with *h*_Au,Ag_ ∼ 19 ± 2 nm annealed for 5 min at 250 °C, and 2 min of MACE. Red outline and red curve, SiNWs with a diameter *d* = 68 ± 18 nm, synthesized using a Au film with *h*_Au_ ∼ 11 ± 1 nm annealed 3 min at 250 °C, and 2 min of MACE. Green outline and green curve, SiNWs with a diameter *d* = 129 ± 28 nm, synthesized using a AuAg film with *h*_Au,Ag_ ∼ 44 ± 5 nm, annealed for 15 min at 250 °C and 6 minutes of MACE. The wavelengths at which the HE_11_ sand HE_12_ modes are expected to occur according to our FDTD simulations (Figure S7) are shown with the respective colored dotted lines. The three colored rectangles next to the reflectance spectra are the corresponding optical microscopy images of these samples. (b) SiNW ethanolic solutions prepared by sonicating Si substrates prepared after 6 min of MACE, same wire morphology and diameter, and color code as in (a). Top, SEM images showing the severed SiNWs, obtained by drying a droplet of the SiNW solution on top of a clean Si piece. Bottom, transmittance spectra of the SiNW solutions. These samples were prepared under the same dewetting-assisted patterning conditions (i.e., identical catalyst film morphology and dimension) as the samples shown in (a).

Some of the VA-SiNW arrays substrates prepared in this work showed bright colors, which can be attributed to leaky guided modes.^4−6,8^ These modes are highly dependent on the nanowire diameter: a 10–15 nm change in diameter can shift these resonances by up to 50 nm.^4−6,8^Figure 4 shows a selection of three nanowire samples prepared via dewetting-assisted patterning and MACE, which have an appropriate wire density, diameter, and narrow size distribution to show defined leaky guided-modes. As expected, the thinnest wires show absorption in the blue region, which red-shifts as the wire diameter increases: the reflectance dip caused by the HE_11_ mode shifts from ca. 380 to 450 nm and 660 nm for wire diameters of *d* = 40 ± 12 nm, *d* = 68 ± 18 nm, and *d* = 129 ± 28 nm, respectively.^4−6,8^ The reflectance spectra of these VA-SiNW arrays was simulated using the finite-difference time-domain (FDTD) method (Figure S7, more details in the experimental section)^4,8^ and confirmed the assignment of the HE_11_ mode for all three samples.^4,8^ The larger SiNW sample (*d* = 129 nm) does not show a resolved HE_12_ mode, expected at around 405 nm according to our simulations (Figure S7). We attribute this to the fairly large size distribution, the highly irregular and noncircular wire cross-section and the relatively low wire density of this sample. Overall these results demonstrate the possibility to synthesize a variety of SiNW random arrays with sub-200 nm wire diameters that can sustain relatively well-defined guided modes, without requiring a complex lithographic approach. Until now, such colored silicon nanowire samples could only be synthesized using subwavelength lithographic approaches, such as electron-beam^6^ or colloidal lithography.^4,29^ Dewetting-assisted patterning provides a simpler alternative that eliminates this requirement.

### Colored SiNW Dispersions

We demonstrate the successful transfer of the SiNWs into ethanolic solutions via sonication (Figure 4b). Because of the random orientation of the SiNW long-axis in solution, both guided modes and Mie resonances can be excited, likely to occur in a similar range of wavelengths.^7^ This is supported by the well-defined transmittance spectra of the SiNW dispersions (Figure 4b), which are quite similar to the reflectance spectra measured on the corresponding solid state VA-SiNW arrays (Figure 4a). This is another demonstration of the relative uniformity of the Si nanostructures that can be produced by combining dewetting-assisted patterning and MACE. SiNW dispersions with small diameters (typically below 20 nm), can be produced via the solution–liquid–solid (SLS) synthesis.^50,51^ However, to our knowledge, the synthesis of solution-dispersible SiNWs exhibiting clearly defined optical resonances that are not attributed to quantum-sized effects has not yet been demonstrated *via* SLS or any alternative method.

### Patterning Black and Colored Silicon via Shadow-Masking (Figure 5)

**Figure 5:**
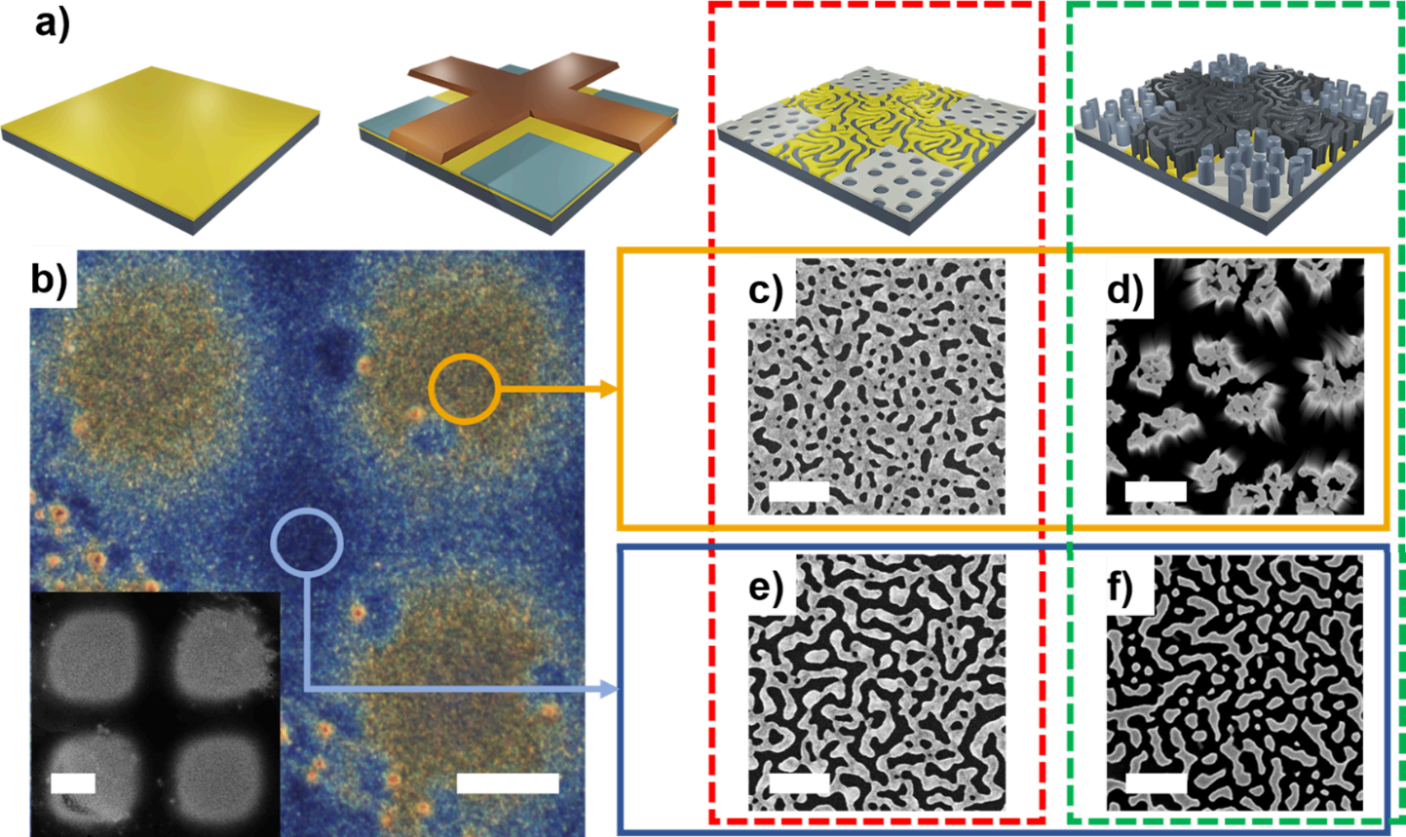
(a) Schematic depiction of the shadow masking approach to pattern Ag micrometer-sized squares on top of the Au-coated Si substrate. From left to right: The process starts with sputtering a serpentine-like porous Au film on top of Si. Afterward, a TEM grid is used as a shadow mask during Ag sputtering. During thermal annealing, the metal film dewets, resulting in two types of regions with different film morphologies. After MACE, areas with two different nanostructured Si morphologies with distinct colors are formed. (b) Optical microscope image of the patterned nanostructured Si substrates, showing the different colored regions. Inset: corresponding low magnification SEM image. Scale bars: 20 μm. (c, e) Representative SEM images of the metal films after thermal annealing: (c) AuAg film regions and (e) Au film regions. (d, f) Representative SEM images obtained after MACE of (d) the SiNWs (holey AuAg regions), and (f) Si nanowalls (serpentine-like porous Au regions). (c–f) Scale bars: 200 nm.

The potential of our approach to prepare color filters was demonstrated using a TEM grid as a shadow mask to pattern regions with a serpentine-like Au film and regions with a porous AuAg film (see Figure S8 for top-view SEM images of the as-sputtered films). After 5 min of annealing at 250 °C, these regions respectively yield: holey films composed of small circular holes (Figure 5c) and porous serpentine-like films (Figure 5e). After MACE, an array of yellow colored SiNW squares (Figure 5d) embedded within a porous bluish silicon grid pattern (Figure 5f) result. The approach is thus compatible with the patterning of micrometer-sized regions with nanoscale features that provide defined optical properties, without requiring advanced lithographic approaches. With further process optimization and the use of appropriate shadow masks and substrates, dewetting-assisted patterning could provide a viable alternative for the fabrication of silicon-based color filters.

## Conclusion

Herein, we report the use of thermal dewetting to pattern Au and AuAg catalytic etching films with tunable dimensions, coverage, and morphology. Successful transfer of these patterns into silicon is demonstrated via MACE to synthesize tubular silicon, Si nanowalls, and VA-SiNW arrays. The influence of sputtering and annealing times and film composition on the resulting nanostructured silicon morphology, coverage, and optical properties is reported. In particular, when combined with MACE, dewetting-assisted patterning can be used to synthesize a variety of nanostructured colored and black silicons. This approach can produce well-defined VA-SiNWs with diameters down to 40 nm, which strongly interact with light by supporting leaky guided-modes, giving rise to vibrant colors. This lithography-free route is fast; dewetting occurs within seconds to minutes; simple and versatile. Combined with shadow masking, dewetting-assisted patterning can be used to pattern regions with different nanostructured silicon morphologies and colors, with potential for the fabrication of Si-based color filters.^6^ Additionally, our approach is compatible with the preparation of SiNW-containing solutions that have enhanced and tunable light absorption properties, which could be deposited on arbitrary substrates, with potential applications for nanowire-based ultrathin solar cells,^52^ photodetectors, photoelectrodes and biosensors.^24,52,53^ As such, this work is a useful addition to the existing methods for synthesizing colored and black nanostructured silicon, relevant to a variety of research fields.

## Supporting Information Available

Additional SEM images of Au/AZO film and the respective SiNWs prepared via MACE, together with the corresponding reflectance spectra; SEM images of Au and Au/Ag films sputtered for different times and of the native Au and AuAg films patterned via shadow-masking; EDS maps of the Au/Ag films after annealing; and simulated reflectance spectra (PDF)

## References

[ref1] Yang D. Introduction. In Handbook of Photovoltaic Silicon; Yang D., Ed.; Springer: Berlin, 2019; pp 1–5.

[ref2] Tilli M.; Haapalinna A. Chapter 1—Properties of silicon. In Handbook of Silicon Based MEMS Materials and Technologies, 3rd ed.; Tilli M., Paulasto-Krockel M., Petzold M., Theuss H., Motooka T., Lindroos V., Eds.; Elsevier: 2020; pp 3-17.

[ref3] Glassner S.; Zeiner C.; Periwal P.; Baron T.; Bertagnolli E.; Lugstein A. Multi-Mode Silicon Nanowire Transistors. Nano Lett. 2014, 14, 6699–6703. 10.1021/nl503476t. 25303290 PMC4245716

[ref4] Wendisch F. J.; Abazari M.; Mahdavi H.; Rey M.; Vogel N.; Musso M.; Diwald O.; Bourret G. R. Morphology-Graded Silicon Nanowire Arrays via Chemical Etching: Engineering Optical Properties at the Nanoscale and Macroscale. ACS Appl. Mater. Interfaces 2020, 12 (11), 13140–13147. 10.1021/acsami.9b21466. 32129591 PMC7082793

[ref5] Bartschmid T.; Wendisch F. J.; Farhadi A.; Bourret G. R. Recent Advances in Structuring and Patterning Silicon Nanowire Arrays for Engineering Light Absorption in Three Dimensions. ACS Appl. Energy Mater. 2022, 5 (5), 5307–5317. 10.1021/acsaem.1c02683. 35647497 PMC9131305

[ref6] Seo K.; Wober M.; Steinvurzel P.; Schonbrun E.; Dan Y.; Ellenbogen T.; Crozier K. B. Multicolored Vertical Silicon Nanowires. Nano Lett. 2011, 11 (4), 1851–1856. 10.1021/nl200201b. 21413684

[ref7] Abujetas D. R.; Paniagua-Dominguez R.; Sánchez-Gil J. A. Unraveling the Janus Role of Mie Resonances and Leaky/Guided Modes in Semiconductor Nanowire Absorption for Enhanced Light Harvesting. ACS Photonics 2015, 2 (7), 921–929. 10.1021/acsphotonics.5b00112.

[ref8] Wang B.; Leu P. W. Tunable and Selective Resonant Absorption in Vertical Nanowires. Opt. Lett. 2012, 37 (18), 3756–3758. 10.1364/OL.37.003756. 23041849

[ref9] Wendisch F. J.; Saller M. S.; Eadie A.; Reyer A.; Musso M.; Rey M.; Vogel N.; Diwald O.; Bourret G. R. Three-Dimensional Electrochemical Axial Lithography on Si Micro- and Nanowire Arrays. Nano Lett. 2018, 18 (11), 7343–7349. 10.1021/acs.nanolett.8b03608. 30359028 PMC6238956

[ref10] Bartschmid T.; Farhadi A.; Musso M. E.; Goerlitzer E. S. A.; Vogel N.; Bourret G. R. Self-Assembled Au Nanoparticle Monolayers on Silicon in Two- and Three-Dimensions for Surface-Enhanced Raman Scattering Sensing. ACS Appl. Nano Mater. 2022, 5 (8), 11839–11851. 10.1021/acsanm.2c01904. 36062062 PMC9425434

[ref11] Tieu T.; Alba M.; Elnathan R.; Cifuentes-Rius A.; Voelcker N. H. Advances in Porous Silicon–Based Nanomaterials for Diagnostic and Therapeutic Applications. Adv. Therap. 2019, 2 (1), 1800095 10.1002/adtp.201800095.

[ref12] Arshavsky-Graham S.; Massad-Ivanir N.; Segal E.; Weiss S. Porous Silicon-Based Photonic Biosensors: Current Status and Emerging Applications. Anal. Chem. 2019, 91 (1), 441–467. 10.1021/acs.analchem.8b05028. 30408950

[ref13] Khorasaninejad M.; Abedzadeh N.; Walia J.; Patchett S.; Saini S. S. Color Matrix Refractive Index Sensors Using Coupled Vertical Silicon Nanowire Arrays. Nano Lett. 2012, 12 (8), 4228–4234. 10.1021/nl301840y. 22823137

[ref14] Meng J.; Cadusch J. J.; Crozier K. B. Detector-Only Spectrometer Based on Structurally Colored Silicon Nanowires and a Reconstruction Algorithm. Nano Lett. 2020, 20 (1), 320–328. 10.1021/acs.nanolett.9b03862. 31829611

[ref15] Wan J.; Kaplan A. F.; Zheng J.; Han X.; Chen Y.; Weadock N. J.; Faenza N.; Lacey S.; Li T.; Guo J.; Hu L. Two Dimensional Silicon Nanowalls for Lithium Ion Batteries. Journal of Materials Chemistry A 2014, 2 (17), 6051–6057. 10.1039/C3TA13546B.

[ref16] Chan C. K.; Peng H.; Liu G.; McIlwrath K.; Zhang X. F.; Huggins R. A.; Cui Y. High-Performance Lithium Battery Anodes Using Silicon Nanowires. Nat. Nanotechnol. 2008, 3, 31 10.1038/nnano.2007.411. 18654447

[ref17] Deng J.; Su Y.; Liu D.; Yang P.; Liu B.; Liu C. Nanowire Photoelectrochemistry. Chemical Reviews 2019, 119 (15), 9221–9259. 10.1021/acs.chemrev.9b00232. 31333018

[ref18] Vijselaar W.; Westerik P.; Veerbeek J.; Tiggelaar R. M.; Berenschot E.; Tas N. R.; Gardeniers H.; Huskens J. Spatial Decoupling of Light Absorption and Catalytic Activity of Ni–Mo-Loaded High-Aspect-Ratio Silicon Microwire Photocathodes. Nat. Energy 2018, 3 (3), 185–192. 10.1038/s41560-017-0068-x.

[ref19] Oh I.; Kye J.; Hwang S. Enhanced Photoelectrochemical Hydrogen Production from Silicon Nanowire Array Photocathode. Nano Lett. 2012, 12 (1), 298 10.1021/nl203564s. 22142272

[ref20] Huo C.; Wang J.; Fu H.; Li X.; Yang Y.; Wang H.; Mateen A.; Farid G.; Peng K.-Q. Metal-Assisted Chemical Etching of Silicon in Oxidizing HF Solutions: Origin, Mechanism, Development, and Black Silicon Solar Cell Application. Advanced Functional Materials 2020, 30 (52), 2005744 10.1002/adfm.202005744.

[ref21] Savin H.; Repo P.; von Gastrow G.; Ortega P.; Calle E.; Garin M.; Alcubilla R. Black Silicon Solar Cells with Interdigitated Back-Contacts Achieve 22.1% Efficiency. Nat. Nanotechnol. 2015, 10 (7), 624–628. 10.1038/nnano.2015.89. 25984832

[ref22] Chai J. Y. H.; Wong B. T.; Juodkazis S. Black-Silicon-Assisted Photovoltaic Cells for Better Conversion Efficiencies: A Review on Recent Research and Development Efforts. Materials Today Energy 2020, 18, 100539 10.1016/j.mtener.2020.100539.

[ref23] Soueiti J.; Sarieddine R.; Kadiri H.; Alhussein A.; Lerondel G.; Habchi R. A Review of Cost-Effective Black Silicon Fabrication Techniques and Applications. Nanoscale 2023, 15 (10), 4738–4761. 10.1039/D2NR06087F. 36808191

[ref24] Jiang Y.; Li X.; Liu B.; Yi J.; Fang Y.; Shi F.; Gao X.; Sudzilovsky E.; Parameswaran R.; Koehler K.; Nair V.; Yue J.; Guo K.; Fang Y.; Tsai H.-M.; Freyermuth G.; Wong R. C. S.; Kao C.-M.; Chen C.-T.; Nicholls A. W.; Wu X.; Shepherd G. M. G.; Tian B. Rational Design of Silicon Structures for Optically Controlled Multiscale Biointerfaces. Nat. Biomed. Eng. 2018, 2 (7), 508–521. 10.1038/s41551-018-0230-1. 30906646 PMC6430241

[ref25] Ballif C.; Perret-Aebi L.-E.; Lufkin S.; Rey E. Integrated Thinking for Photovoltaics in Buildings. Nature Energy 2018, 3 (6), 438–442. 10.1038/s41560-018-0176-2.

[ref26] Jiang C.; Zhang G.; Hong Z.; Chen J.; Li Y.; Yuan X.; Lin Y.; Yu C.; Wang T.; Song T.; Wang Y.; Sun B. Colored Silicon Heterojunction Solar Cells Exceeding 23.5% Efficiency Enabled by Luminescent Down-Shift Quantum Dots. Adv. Mater. 2023, 35 (6), 2208042 10.1002/adma.202208042. 36433769

[ref27] Morales A. M.; Lieber C. M. A Laser Ablation Method for the Synthesis of Crystalline Semiconductor Nanowires. Science 1998, 279 (5348), 208–211. 10.1126/science.279.5348.208. 9422689

[ref28] Fu Y. Q.; Colli A.; Fasoli A.; Luo J. K.; Flewitt A. J.; Ferrari A. C.; Milne W. I. Deep Reactive Ion Etching as a Tool for Nanostructure Fabrication. Journal of Vacuum Science & Technology B: Microelectronics and Nanometer Structures 2009, 27 (3), 1520 10.1116/1.3065991.

[ref29] Wendisch F. J.; Rey M.; Vogel N.; Bourret G. R. Large-Scale Synthesis of Highly Uniform Silicon Nanowire Arrays Using Metal-Assisted Chemical Etching. Chem. Mater. 2020, 32 (21), 9425–9434. 10.1021/acs.chemmater.0c03593. 33191979 PMC7659364

[ref30] Alhmoud H.; Brodoceanu D.; Elnathan R.; Kraus T.; Voelcker N. H. A MACEing Silicon: Towards Single-Step Etching of Defined Porous Nanostructures for Biomedicine. Progress in Materials Science 2021, 116, 100636 10.1016/j.pmatsci.2019.100636.

[ref31] Chen W.; Yu L.; Misra S.; Fan Z.; Pareige P.; Patriarche G.; Bouchoule S.; Cabarrocas P. R. i. Incorporation and Redistribution of Impurities into Silicon Nanowires During Metal-Particle-Assisted Growth. Nat. Commun. 2014, 5, 4134 10.1038/ncomms5134. 24920212

[ref32] Zhang X.; Lin J. K.; Wickramanayaka S.; Zhang S.; Weerasekera R.; Dutta R.; Chang K. F.; Chui K.-J.; Li H. Y.; Wee Ho D. S.; Ding L.; Katti G.; Bhattacharya S.; Kwong D.-L. Heterogeneous 2.5D Integration on through Silicon Interposer. Applied Physics Reviews 2015, 2 (2), 021308 10.1063/1.4921463.

[ref33] Srivastava R. P.; Khang D.-Y. Structuring of Si into Multiple Scales by Metal-Assisted Chemical Etching. Adv. Mater. 2021, 33 (47), 2005932 10.1002/adma.202005932. 34013605

[ref34] Lai R. A.; Hymel T. M.; Narasimhan V. K.; Cui Y. Schottky Barrier Catalysis Mechanism in Metal-Assisted Chemical Etching of Silicon. ACS Appl. Mater. Interfaces 2016, 8 (14), 8875–8879. 10.1021/acsami.6b01020. 27018712

[ref35] Wendisch F. J.; Oberreiter R.; Salihovic M.; Elsaesser M. S.; Bourret G. R. Confined Etching within 2D and 3D Colloidal Crystals for Tunable Nanostructured Templates: Local Environment Matters. ACS Appl. Mater. Interfaces 2017, 9 (4), 3931–3939. 10.1021/acsami.6b14226. 28094914

[ref36] Rey M.; Wendisch F. J.; Goerlitzer E. S. A.; Tang J. S. J.; Bader R. S.; Bourret G. R.; Vogel N. Anisotropic Silicon Nanowire Arrays Fabricated by Colloidal Lithography. Nanoscale Adv. 2021, 3 (12), 3634–3642. 10.1039/D1NA00259G. 34212129 PMC8204746

[ref37] Wendisch F. J.; Abazari M.; Werner V.; Barb H.; Rey M.; Goerlitzer E. S. A.; Vogel N.; Mahdavi H.; Bourret G. R. Spatioselective Deposition of Passivating and Electrocatalytic Layers on Silicon Nanowire Arrays. ACS Appl. Mater. Interfaces 2020, 12 (47), 52581–52587. 10.1021/acsami.0c14013. 33169967 PMC7705884

[ref38] Romano L.; Vila-Comamala J.; Jefimovs K.; Stampanoni M. High-Aspect-Ratio Grating Microfabrication by Platinum-Assisted Chemical Etching and Gold Electroplating. Advanced Engineering Materials 2020, 22 (10), 2000258 10.1002/adem.202000258.

[ref39] Tang J. S. J.; Bader R. S.; Goerlitzer E. S. A.; Wendisch J. F.; Bourret G. R.; Rey M.; Vogel N. Surface Patterning with SiO_2_@PNiPAm Core-Shell Particles. ACS Omega 2018, 3 (9), 12089–12098. 10.1021/acsomega.8b01985. 30288467 PMC6166996

[ref40] Thompson C. V. Solid-State Dewetting of Thin Films. Annual Review of Materials Research 2012, 42 (1), 399–434. 10.1146/annurev-matsci-070511-155048.

[ref41] Tesler A. B.; Maoz B. M.; Feldman Y.; Vaskevich A.; Rubinstein I. Solid-State Thermal Dewetting of Just-Percolated Gold Films Evaporated on Glass: Development of the Morphology and Optical Properties. The Journal of Physical Chemistry C 2013, 117 (21), 11337–11346. 10.1021/jp400895z.

[ref42] Altomare M.; Nguyen N. T.; Schmuki P. Templated Dewetting: Designing Entirely Self-Organized Platforms for Photocatalysis. Chemical Science 2016, 7 (12), 6865–6886. 10.1039/C6SC02555B. 28567258 PMC5450593

[ref43] Azeredo B. P.; Sadhu J.; Ma J.; Jacobs K.; Kim J.; Lee K.; Eraker J. H.; Li X.; Sinha S.; Fang N.; Ferreira P.; Hsu K. Silicon Nanowires with Controlled Sidewall Profile and Roughness Fabricated by Thin-Film Dewetting and Metal-Assisted Chemical Etching. Nanotechnology 2013, 24 (22), 225305 10.1088/0957-4484/24/22/225305. 23644697

[ref44] Kong L.; Chiam S. Y.; Chim W. K. Metal-Assisted Silicon Chemical Etching Using Self-Assembled Sacrificial Nickel Nanoparticles Template for Antireflection Layers in Photovoltaic and Light-Trapping Devices. ACS Appl. Nano Mater. 2019, 2 (11), 7025–7031. 10.1021/acsanm.9b01528.

[ref45] Stafiniak A.; Prażmowska J.; Macherzyński W.; Paszkiewicz R. Nanostructuring of Si Substrates by a Metal-Assisted Chemical Etching and Dewetting Process. RSC Advances 2018, 8 (54), 31224–31230. 10.1039/C8RA03711F. 35548763 PMC9085574

[ref46] Palik E. D., Ed. Handbook of Optical Constants of Solids; Elsevier.

[ref47] Rey M.; Elnathan R.; Ditcovski R.; Geisel K.; Zanini M.; Fernandez-Rodriguez M.-A.; Naik V. V.; Frutiger A.; Richtering W.; Ellenbogen T.; Voelcker N. H.; Isa L. Fully Tunable Silicon Nanowire Arrays Fabricated by Soft Nanoparticle Templating. Nano Lett. 2016, 16 (1), 157–163. 10.1021/acs.nanolett.5b03414. 26672801

[ref48] Romano L.; Vila-Comamala J.; Jefimovs K.; Stampanoni M. Effect of Isopropanol on Gold Assisted chemical Etching of Silicon Microstructures. Microelectron. Eng. 2017, 177, 59–65. 10.1016/j.mee.2017.02.008.

[ref49] Su D.; Yu M.; Zhang G.; Jiang S.; Qin Y.; Li M.-y. Highly Thermally Stable Au–Al Bimetallic Conductive Thin Films with a Broadband Transmittance Between UV and NIR Regions. Journal of Materials Chemistry C 2020, 8 (8), 2852–2860. 10.1039/C9TC06496F.

[ref50] Heitsch A. T.; Fanfair D. D.; Tuan H.-Y.; Korgel B. A. Solution–Liquid–Solid (SLS) Growth of Silicon Nanowires. J. Am. Chem. Soc. 2008, 130 (16), 5436–5437. 10.1021/ja8011353. 18373344

[ref51] Wang F.; Dong A.; Buhro W. E. Solution–Liquid–Solid Synthesis, Properties, and Applications of One-Dimensional Colloidal Semiconductor Nanorods and Nanowires. Chemical Reviews 2016, 116 (18), 10888–10933. 10.1021/acs.chemrev.5b00701. 26974736

[ref52] Kim S.-K.; Day R. W.; Cahoon J. F.; Kempa T. J.; Song K.-D.; Park H.-G.; Lieber C. M. Tuning Light Absorption in Core/Shell Silicon Nanowire Photovoltaic Devices through Morphological Design. Nano Lett. 2012, 12 (9), 4971–4976. 10.1021/nl302578z. 22889329

[ref53] Baraban L.; Ibarlucea B.; Baek E.; Cuniberti G. Hybrid Silicon Nanowire Devices and Their Functional Diversity. Advanced Science 2019, 6 (15), 1900522 10.1002/advs.201900522. 31406669 PMC6685480

