# Supplementary material for: Dewetting-Assisted Patterning: A Lithography-Free Route to Synthesize Black and Colored Silicon
Source: ACS Appl Mater Interfaces. 2023 Sep 5;15(37):44087–96. doi: 10.1021/acsami.3c08533 (PMC10520913; doi:10.1021/acsami.3c08533)
Supplement: Supplementary file 1 — am3c08533_si_001.pdf [file am3c08533_si_001.pdf]

Supporting Information

Dewetting-Assisted Patterning: A Lithography-Free Route to Synthesize Black and Colored Silicon

Amin Farhadi, Theresa Bartschmid and Gilles R. Bourret*

Department of Chemistry and Physics of Materials, University of Salzburg, Jakob Haringerstraße 2a, A-5020 Salzburg, Austria

*E-mail: gilles.bourret@plus.ac.at

Keywords: metal-assisted chemical etching, silicon nanowires, dewetting, nanostructured silicon, black silicon, colored silicon

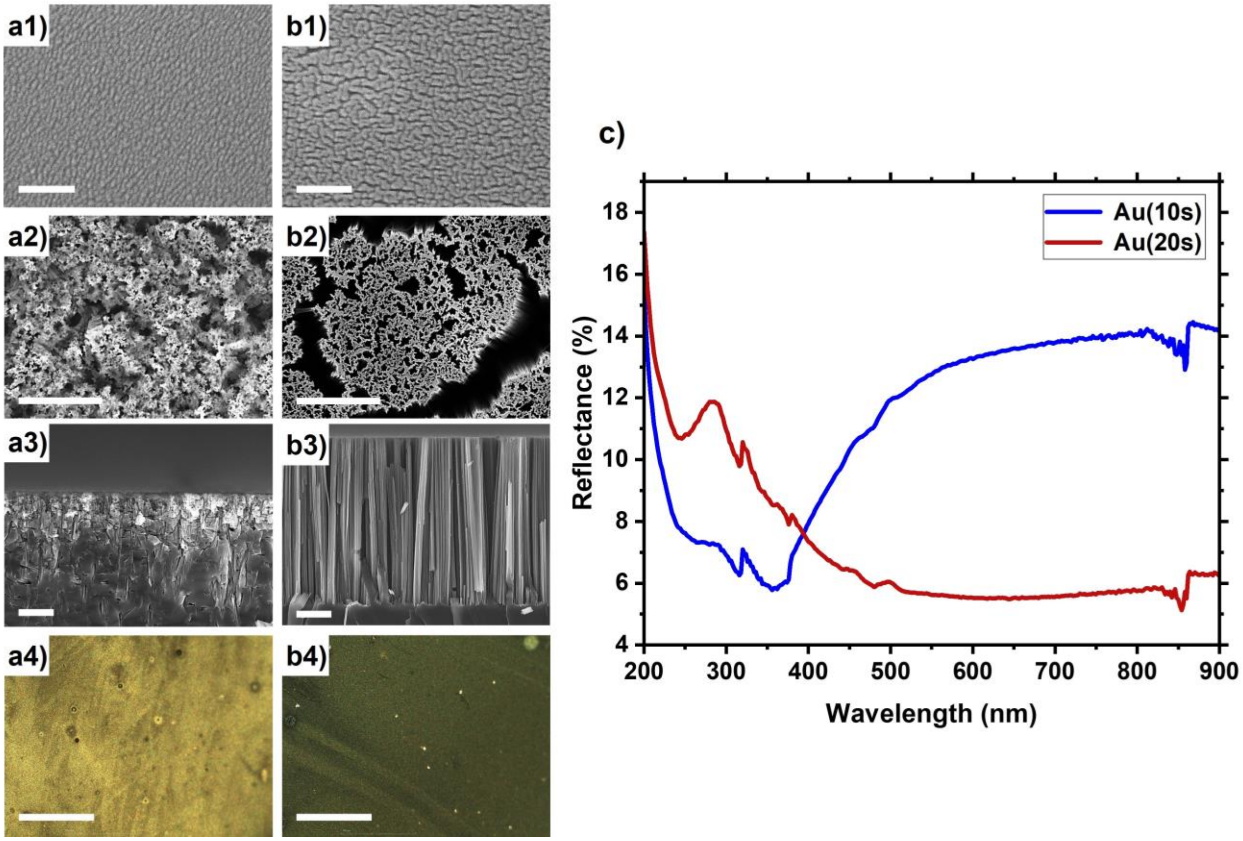

Figure S1. Effect of Au film thickness on AZO/Si: 10s sputtering (a1-a4) and 20s sputtering (b1-b4). SEM images of: (a1, b1) the Au film after sputtering, scale bars: 100 nm; Si after MACE, (a2, b2) top view, and (a3, b3) cross-section, scale bars: 1 µm. (a4, b4) Optical microscope images capturing the realistic color of the nanostructured silicon (scale bars: 100 µm). c) Reflectance spectra of porous Si fabricated from 10s of gold sputtering (blue curve) and Si nanowalls fabricated from 20s of gold sputtering (red curve).

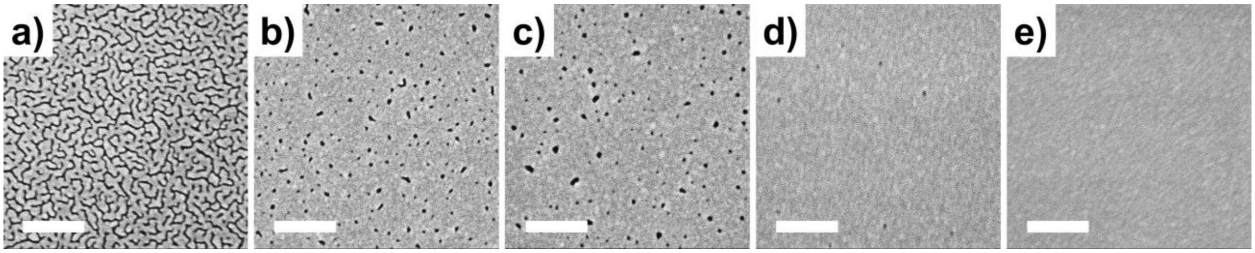

Figure S2. Effect of Au sputtering time on Au film morphology before thermal dewetting. The resulted gold film sputtered for a) 50 s, b) 60 s, c) 67 s, d) 70s, e) 80 s. Scale bars: 200 nm.

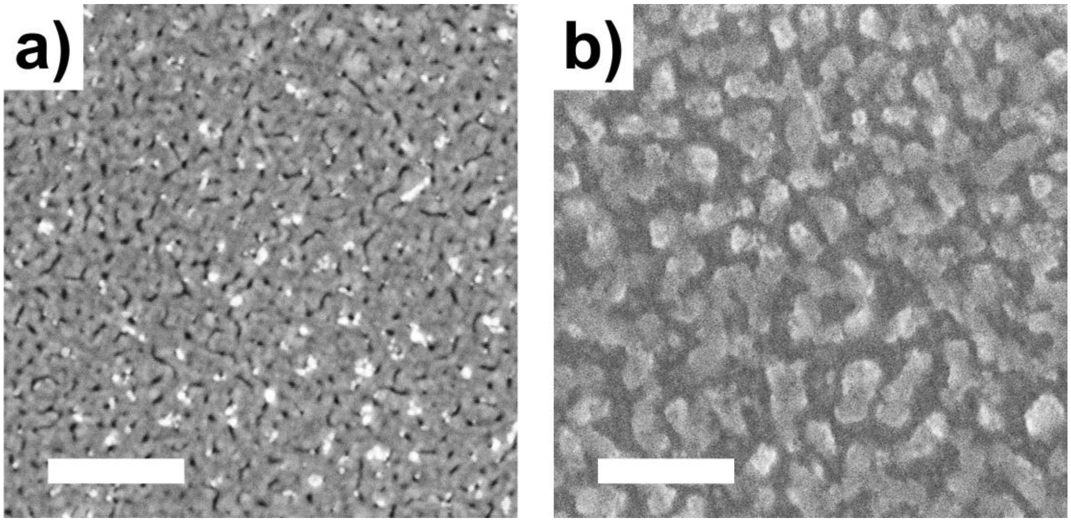

Figure S3. Effect of sputtering time on Au-Ag film morphology before thermal dewetting. The resulted bimetallic film sputtered for a) 50 s of Au followed by 50 s of Ag sputtering, b) 70 s of Au followed by 70 s of Ag sputtering. Scale bars: 200 nm.

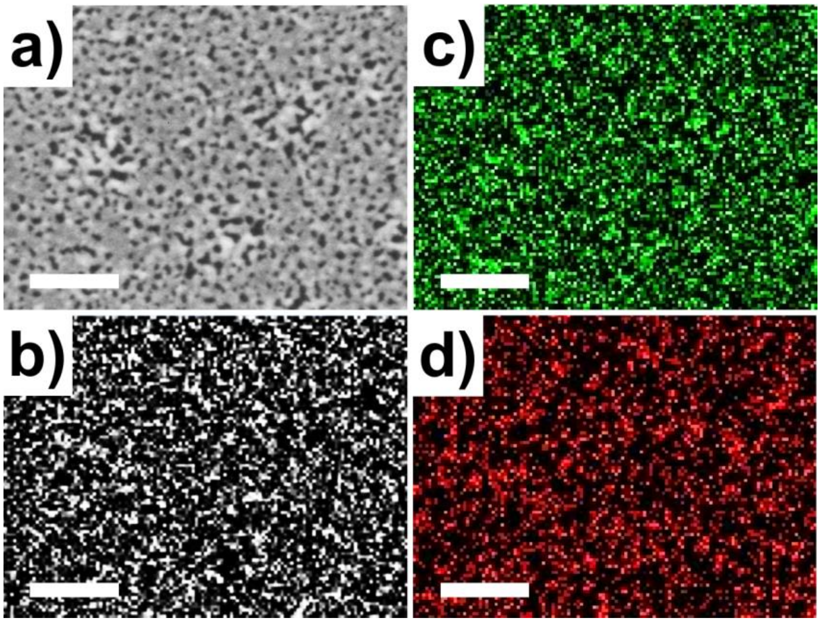

Figure S4. EDS elemental mapping of the of the Au-Ag alloy film sputtered for 50s for each element and then annealed for 5 min. a) In-lens SEM image of the analyzed area. b) Si distribution shown in white, c) Au distribution shown in green and d) Ag distribution shown in red. Scale bars: 500 nm.

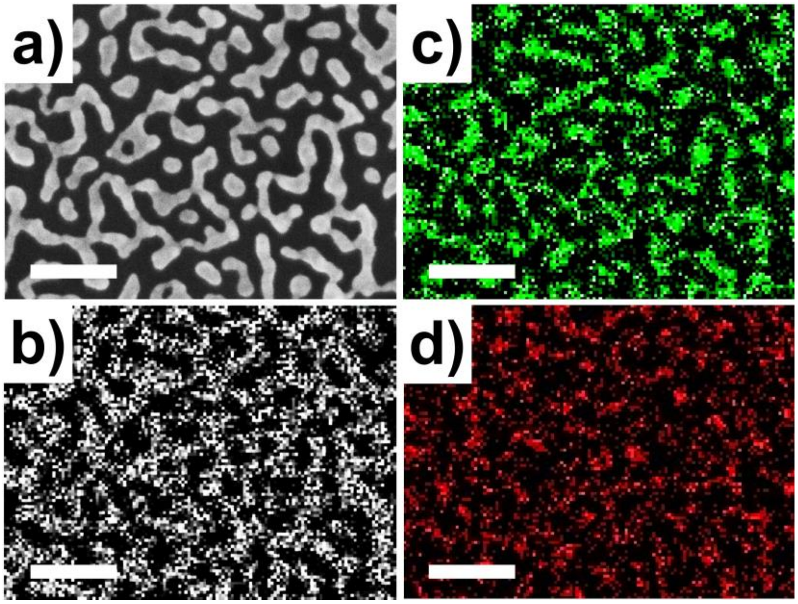

Figure S5. EDS elemental mapping of the of the Au-Ag alloy film sputtered for 50s for each element and then annealed for 30 min. a) In-lens SEM image of the analyzed area. b) Si distribution shown in white, c) Au distribution shown in green and d) Ag distribution shown in red. Scale bars: 500 nm.

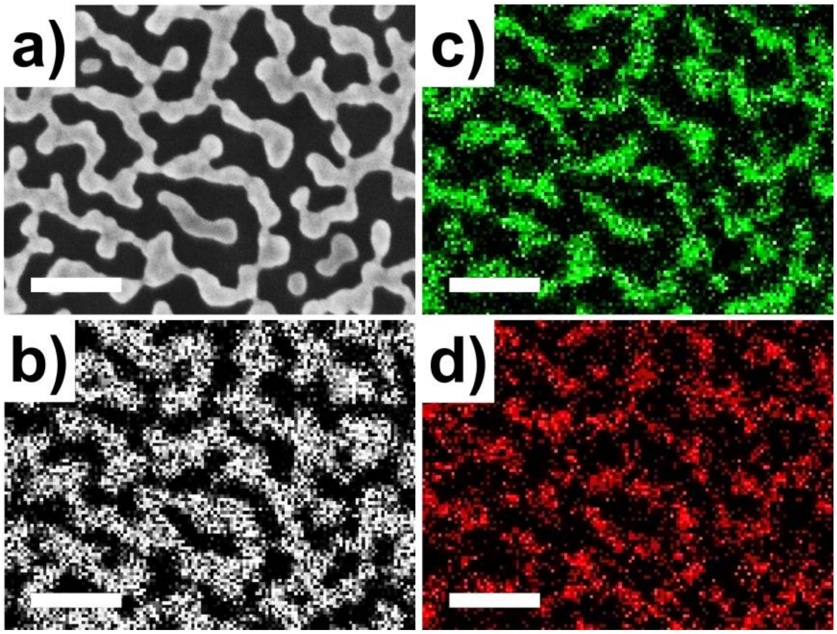

Figure S6. EDS elemental mapping of the of the Au-Ag alloy film sputtered for 70s for each element and then annealed for 30 min a) In-lens SEM image of the analyzed area. b) Si distribution shown in white, c) Au distribution shown in green and d) Ag distribution shown in red. Scale bars: 500 nm.

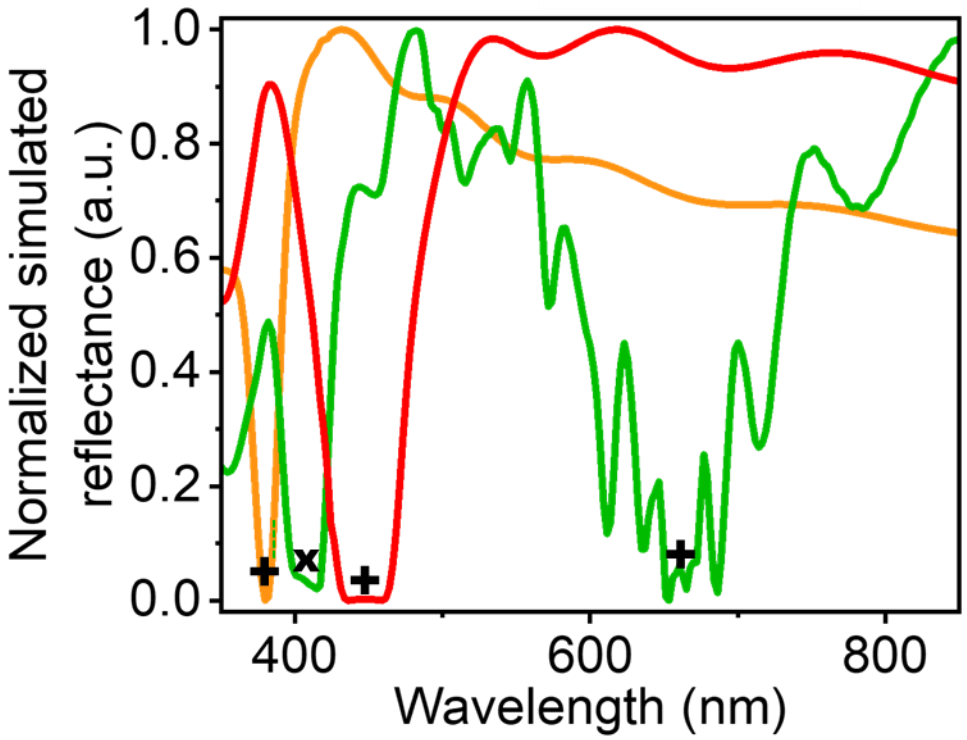

Figure S7. Reflectance spectra of VA-SiNW arrays simulated using the finite-difference time-domain (FDTD) method. Tapered wires (see experimental section for details) were simulated in a hexagonal array (pitch: 480 nm and length: 1.5 µm), with the following nominal diameters: 40 nm orange curve), 68 nm (red curve) and 129 nm (green curve). The "+" signs show the location of the corresponding HE_{11} modes, and the "x" sign locates the expected HE_{12} mode for the large nanowire sample (d: 129 nm, green curve).

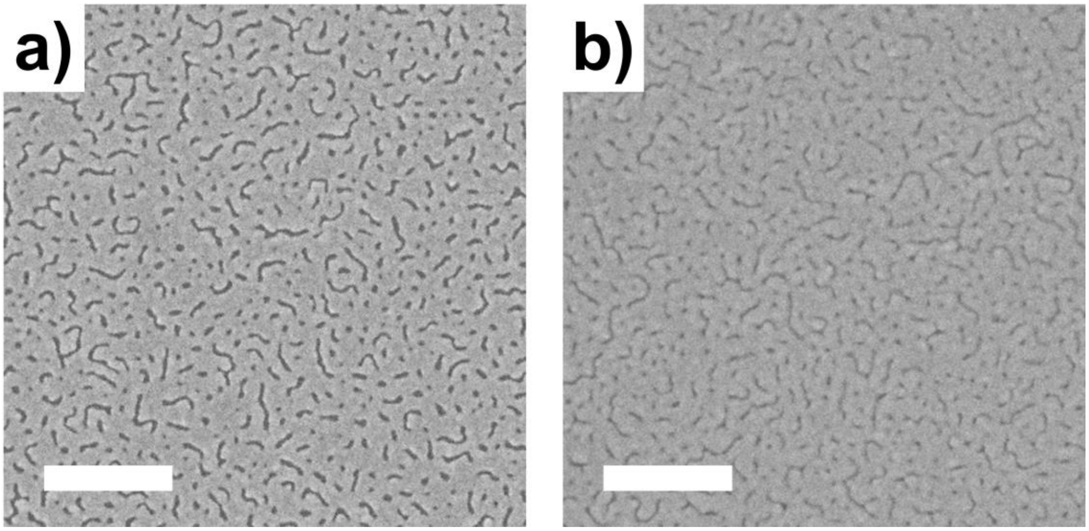

Figure S8. Representative top-view SEM images of the as-sputtered Au and AuAg regions (e.g. before annealing) patterned via shadow masking. a) Au film only. b) AuAg film. Scale bars: 200 nm
